# Testicular ergonomics: whole-body vibration, mechanotransduction, and male reproductive health in heavy machinery operators

**DOI:** 10.3389/fpubh.2026.1947463

**Published:** 2026-09-29

**Authors:** Bogdan Doroftei, Ahmed Adel Mansour Kamar, Alin Ciobica, Dragos Puia, Mara Doroftei, David Puha, Bogdan Puha

**Affiliations:** 1 Origyn Fertility Center, Iași, Romania; 2 Faculty of Medicine, University of Medicine and Pharmacy, “Grigore T. Popa”, Iași, Romania; 3 Spitalul Clinic de Obstetrică și Ginecologie “Cuza Vodă”, Iași, Romania; 4 Department Biology, Faculty of Biology, Doctoral School, Alexandru Ioan Cuza University, Iași, Romania; 5 Department Biology, Faculty of Biology, Alexandru Ioan Cuza University (UAIC), Iași, Romania; 6 Department of Urology, “Dr. C.I. Parhon” Clinical Hospital, Iași, Romania; 7 Sf. Spiridon Emergency Clinical Hospital of Iasi, Iași, Romania; 8 Department of Orthopedics and Traumatology, "Grigore T. Popa" University of Medicine and Pharmacy, Iași, Romania

**Keywords:** heavy machinery operators, male fertility, male reproductive health, mechanotransduction, occupational exposure, oxidative stress, spermatogenesis, testicular ergonomics

## Abstract

Whole-body vibration (WBV) is a common occupational exposure among heavy machinery operators and has traditionally been investigated in relation to musculoskeletal, spinal, and neurological disorders. However, its potential impact on male reproductive health remains insufficiently characterized. This narrative review critically evaluates current occupational, experimental, and mechanobiological evidence concerning the potential association between chronic WBV exposure and male reproductive dysfunction. Particular attention is given to mechanotransduction and its potential interaction with oxidative stress, mitochondrial dysfunction, microvascular alterations, blood-testis barrier disruption, inflammation, endocrine alterations, and impaired spermatogenesis. Available evidence suggests biological plausibility but remains heterogeneous and insufficient to establish a causal relationship between occupational WBV and male infertility. We introduce the concept of Testicular Ergonomics as a hypothesis-generating framework for integrating male reproductive health into occupational vibration research and prevention. This framework emphasizes improved vibration exposure assessment, engineering and administrative controls, and standardized reproductive-health evaluation while accounting for relevant occupational and lifestyle confounders. Future prospective human and mechanistic studies integrating standardized WBV measurements with reproductive and molecular outcomes are required to determine whether chronic occupational vibration represents an independent risk to male reproductive health and to inform evidence-based preventive strategies.

## 1 Introduction

Whole-body vibration (WBV) is a common occupational exposure among operators of heavy machinery, commercial vehicles, agricultural equipment, and industrial transport systems. Mechanical oscillations generated by vehicles or machinery are transmitted through the seat or supporting surface to the body, with the biological effects influenced by vibration magnitude, frequency, direction, exposure duration, posture, and workplace ergonomics. Research on WBV has traditionally focused on musculoskeletal disorders, particularly low back pain, together with neurological and vascular complications. In contrast, its potential effects on male reproductive health remain relatively understudied (1–12).

The testes are continuously exposed to mechanical forces generated by body movement, posture, and external loading. Normal testicular function depends on the coordinated interaction of germ cells, Sertoli cells, Leydig cells, the blood-testis barrier (BTB), and the testicular microvasculature, which together maintain spermatogenesis and endocrine homeostasis (13–18). Although these structures are highly regulated, little attention has been given to how chronic occupational vibration may influence their function.

Evidence directly linking WBV to male reproductive dysfunction is limited but growing. In an occupational study, taxi drivers chronically exposed to WBV demonstrated reduced semen quality compared with non-exposed controls (19). Occupational WBV has also been examined in relation to alterations in male sex hormone levels (20), while recent occupational research has examined WBV alongside other workplace exposures in relation to male fertility indicators (21). Experimental studies have also shown that prolonged WBV alters reproductive physiology in animal models, affecting spermatid production, endocrine function, oxidative balance, and inflammatory pathways (22). Earlier reports suggested that occupational vibration may adversely affect male reproductive function (23), while a recent systematic review identified vibration as a potential occupational risk factor for reproductive health in both men and women (24). Although these findings support further investigation, the available evidence remains heterogeneous and insufficient to establish causality.

One possible explanation for these observations is mechanotransduction, the process by which cells convert mechanical stimuli into biochemical signals (25–29). Mechanical loading can alter cytoskeletal organization, extracellular matrix interactions, and intracellular signaling, leading to changes in cellular function. More recently, mechanosensitive PIEZO ion channels have emerged as important mediators of cellular responses to mechanical forces (30). In addition, experimental evidence has shown that increased extracellular matrix stiffness disrupts stem Leydig-cell homeostasis, increases mitochondrial reactive oxygen species production through PIEZO1-dependent signaling, and reduces testosterone synthesis (31). Although these findings were not obtained from WBV models, they support the concept that mechanical stimuli can directly influence testicular biology.

Mechanical stress may also activate downstream pathways that are already recognized contributors to male reproductive dysfunction. Oxidative stress, mitochondrial dysfunction, inflammation, and impaired antioxidant defenses have been implicated in sperm damage and impaired fertility (32–35). Because spermatozoa contain abundant polyunsaturated fatty acids and have limited intrinsic antioxidant capacity, they are particularly susceptible to oxidative injury. Excessive reactive oxygen species may impair sperm motility, membrane integrity, DNA stability, and overall reproductive function (32–38). Vibration-induced vascular changes may further compromise testicular perfusion and microcirculatory regulation, potentially affecting the local environment required for normal spermatogenesis (4, 16, 17).

The BTB may represent another vulnerable structure. Formed by specialized junctions between adjacent Sertoli cells, the BTB regulates molecular exchange within the seminiferous epithelium and protects developing germ cells from immune-mediated injury (13–15). Whether chronic WBV disrupts BTB integrity has not been established, but mechanical loading and oxidative stress provide biologically plausible mechanisms that warrant investigation.

Current occupational guidelines for WBV primarily address musculoskeletal and neurological outcomes, while reproductive health is rarely considered in exposure assessment or worker surveillance (2, 6–11). Similarly, important questions remain regarding exposure thresholds, vibration frequency, cumulative dose, reversibility of injury, and interactions with other occupational and lifestyle factors. Future human studies should also adopt standardized methods for semen analysis, including standardized sample collection and assessment of semen volume, sperm concentration, total sperm number, motility, vitality, and morphology according to the WHO Laboratory Manual to improve comparability between investigations (39).

This narrative review summarizes the current evidence linking occupational WBV with male reproductive health and discusses the potential roles of mechanotransduction, oxidative stress, mitochondrial dysfunction, microvascular alterations, BTB integrity, endocrine regulation, and impaired spermatogenesis. We also introduce the concept of Testicular Ergonomics, which emphasizes the integration of male reproductive health into occupational vibration research, workplace design, and preventive strategies. Rather than proposing a new disease entity, this concept provides a framework for considering reproductive outcomes alongside the established health effects of occupational WBV and highlights priorities for future research.

### 1.1 Research question

Can mechanotransduction provide a biological explanation for the potential association between occupational whole-body vibration and male reproductive dysfunction?

## 2 Methodology and literature search strategy

This narrative review summarizes the current evidence on the relationship between occupational WBV and male reproductive health, with particular emphasis on mechanotransduction and its potential role in testicular dysfunction.

The literature search was conducted in PubMed/MEDLINE, Scopus, Web of Science, and Google Scholar for English-language publications from database inception to July 2026. A structured narrative search strategy was used to improve transparency and reproducibility while retaining the narrative scope of the review. Search terms included combinations of “whole-body vibration,” “occupational vibration,” “mechanical vibration,” “male fertility,” “male infertility,” “testis,” “testicular function,” “spermatogenesis,” “semen quality,” “mechanotransduction,” “PIEZO,” “oxidative stress,” “blood-testis barrier,” “Leydig cells,” and “occupational health.” Search terms were combined using the Boolean operators “AND” and “OR” to identify literature linking WBV exposure with reproductive, endocrine, oxidative, vascular, and mechanotransduction-related outcomes.

Peer-reviewed original research articles, systematic reviews, narrative reviews, experimental animal studies, occupational guidelines, and landmark mechanobiology publications were considered. Reference lists of relevant articles were also screened to identify additional studies. Publications focusing exclusively on hand-arm vibration or non-occupational vibration exposure were excluded unless they provided mechanistic insights relevant to WBV-induced biological responses. Priority was given to human occupational evidence, followed by experimental reproductive studies and mechanistic evidence relevant to vibration-induced testicular injury.

Approximately 124 publications were screened, and 51 sources were included based on their relevance to occupational WBV, male reproductive outcomes, or mechanistic pathways. Duplicate records and studies lacking occupational, reproductive, or mechanistic relevance were excluded. Owing to the limited and heterogeneous nature of the available evidence, the findings were synthesized narratively rather than through quantitative analysis. Evidence was organized according to study type, population or experimental model, WBV exposure characteristics, reproductive or biological outcomes, proposed mechanisms, and key methodological limitations.

## 3 Occupational whole-body vibration: exposure and biological basis

### 3.1 What is whole-body vibration?

Whole-body vibration (WBV) refers to mechanical oscillations transmitted to the human body through a supporting surface, most commonly a vehicle seat, floor, or platform. In occupational settings, WBV primarily affects workers who spend prolonged periods operating mobile machinery or driving vehicles, resulting in repeated exposure to low-frequency mechanical vibrations (2, 6–11). The magnitude of the biological response depends on multiple factors, including vibration frequency, acceleration, direction, duration of exposure, posture, body mass, seat characteristics, and individual susceptibility (2, 11).

International standards classify WBV as an important occupational physical hazard and provide standardized methods for measuring and evaluating vibration exposure. The International Organization for Standardization (ISO 2631-1) recommends assessing vibration according to its frequency-weighted acceleration, exposure duration, and transmission through different body axes, allowing comparisons across occupational environments (11). Accordingly, future reproductive-health studies should report WBV magnitude, frequency-weighted acceleration, exposure duration, and measurement axis using standardized ISO 2631-1 procedures to facilitate comparison between studies. These measurements are widely used in ergonomics and occupational health to estimate health risks and guide preventive interventions (2, 11). For occupational risk assessment, daily WBV exposure is commonly expressed as A(8), standardized to an eight-hour reference period. Under European Directive 2002/44/EC, the daily WBV exposure action value is 0.5 m/s^2^ A(8), while the exposure limit value is 1.15 m/s^2^ A(8) (40, 41).

Occupational WBV is encountered across numerous industries. Professional drivers of trucks, buses, taxis, agricultural tractors, forklifts, construction equipment, mining vehicles, and military transport are among the most frequently exposed groups because of prolonged sitting combined with continuous transmission of mechanical vibrations from vehicles and uneven terrain (6–10). Daily exposure often extends over several hours and may continue for many years, increasing cumulative mechanical loading on multiple organ systems (2, 6–10).

Research on WBV has historically focused on its effects on the musculoskeletal system. Numerous epidemiological studies have demonstrated associations between long-term WBV exposure and low back pain, lumbar disc degeneration, spinal disorders, neck pain, and reduced physical function (6–10). Beyond the musculoskeletal system, chronic WBV exposure has also been associated with neurological symptoms, impaired balance, fatigue, peripheral vascular alterations, and reduced work performance, highlighting its multisystem impact (1, 3–10).

Despite extensive investigation of these established health outcomes, the potential effects of WBV on male reproductive function have received comparatively little attention. This gap is noteworthy because the testes are highly vascularized organs with tightly regulated cellular architecture that may be susceptible to chronic mechanical loading and its downstream biological consequences. While direct evidence remains limited, emerging experimental and occupational studies suggest that prolonged WBV may influence pathways involved in testicular homeostasis, providing a rationale for further investigation (19, 22–24).

### 3.2 Occupational exposure to whole-body vibration

Occupational exposure to WBV varies considerably according to vehicle type, machinery characteristics, work environment, and daily exposure time. Professional drivers, heavy-equipment operators, agricultural workers, miners, and construction personnel represent the largest occupational groups exposed to sustained vibration throughout the working day (6–10). Exposure is influenced not only by machinery design but also by road or terrain conditions, driving speed, maintenance of suspension systems, and ergonomic characteristics of the seat and workstation (2, 11).

Unlike acute mechanical trauma, WBV represents a chronic physical exposure in which relatively low-intensity vibrations are repeatedly transmitted to the body over months or years. This cumulative exposure may contribute to progressive biological adaptations or tissue injury that are not immediately apparent clinically (2, 6–10). Consequently, occupational risk assessment emphasizes cumulative vibration dose rather than isolated exposure events.

Current occupational guidelines primarily aim to reduce the risk of musculoskeletal disorders through engineering controls, equipment maintenance, ergonomic seat design, and limitation of daily vibration exposure (2, 6–11). However, reproductive health outcomes are rarely included in routine occupational surveillance or WBV risk assessment, reflecting the limited evidence currently available in this field.

### 3.3 Established biological effects of whole-body vibration

The biological effects of WBV extend beyond the musculoskeletal system. Experimental and clinical studies indicate that chronic vibration exposure may influence multiple physiological systems through mechanical loading, altered tissue perfusion, oxidative stress, inflammatory responses, and neurovascular dysfunction (1, 3–5, 32–35). Although the magnitude of these effects depends on exposure characteristics, they support the concept that WBV produces systemic rather than exclusively local biological responses.

One mechanism receiving increasing attention is mechanotransduction, whereby mechanical stimuli are converted into intracellular biochemical signals that regulate cellular behavior (25–29). Mechanical forces can modify cytoskeletal organization, extracellular matrix interactions, and downstream signaling pathways, ultimately influencing cell survival, metabolism, inflammation, and tissue remodeling. Similar mechanotransductive processes have been implicated in cardiovascular biology, skeletal muscle, cartilage, and bone adaptation, suggesting that mechanically sensitive tissues throughout the body may respond to chronic vibration exposure (25–29).

Mechanical loading has also been associated with increased oxidative stress and mitochondrial dysfunction in several experimental models (32–35). Excessive production of reactive oxygen species may impair cellular homeostasis by promoting lipid peroxidation, protein oxidation, DNA damage, and inflammatory signaling. Because oxidative stress is a recognized contributor to male reproductive dysfunction, these observations provide a biologically plausible link between chronic WBV exposure and impaired testicular function, although direct mechanistic evidence remains limited (32–38).

Collectively, these findings suggest that WBV should be considered a multisystem occupational exposure with potential biological effects extending beyond the traditionally recognized musculoskeletal outcomes. Whether these mechanisms contribute to clinically meaningful impairment of male reproductive health remains an important area for future investigation.

## 4 Potential mechanisms linking whole-body vibration to male reproductive dysfunction

Although the relationship between occupational whole-body vibration (WBV) and male reproductive dysfunction has not been fully established, several biological mechanisms provide a plausible basis for this association. Chronic mechanical loading has been shown to influence cellular signaling, oxidative balance, vascular function, and tissue homeostasis in multiple organ systems (3–10, 25–29, 32–35). Because normal testicular function depends on coordinated interactions among germ cells, Sertoli cells, Leydig cells, the blood-testis barrier (BTB), and an extensive microvascular network, disruption of any of these components could potentially impair spermatogenesis and endocrine function (13–18). The following sections summarize the principal mechanisms that may contribute to vibration-induced testicular dysfunction.

### 4.1 Mechanotransduction

Mechanotransduction refers to the process by which cells detect mechanical stimuli and convert them into intracellular biochemical signals that regulate cellular behavior (25–29). This process is fundamental to tissue adaptation under physiological conditions and contributes to cellular responses in bone, cartilage, skeletal muscle, vascular endothelium, and other mechanically active tissues (25–28).

Mechanical forces are transmitted through the extracellular matrix to integrins, focal adhesions, and the cytoskeleton, initiating signaling cascades that influence gene expression, cellular metabolism, proliferation, apoptosis, and inflammatory responses (25–29). These signaling pathways enable tissues to adapt to changes in their mechanical environment but may also contribute to pathological remodeling when mechanical loading becomes excessive or prolonged.

Although mechanotransduction has been extensively investigated in musculoskeletal biology, its role in testicular physiology remains comparatively unexplored. The testes are continuously subjected to mechanical forces generated by body movement, posture, and external loading, while maintaining a highly organized cellular architecture required for spermatogenesis. Consequently, chronic WBV may alter mechanosensitive signaling within the testicular microenvironment, although direct experimental evidence remains limited. The proposed biological mechanisms linking occupational whole-body vibration to male reproductive dysfunction are summarized in Figure 1.

**Figure 1:**
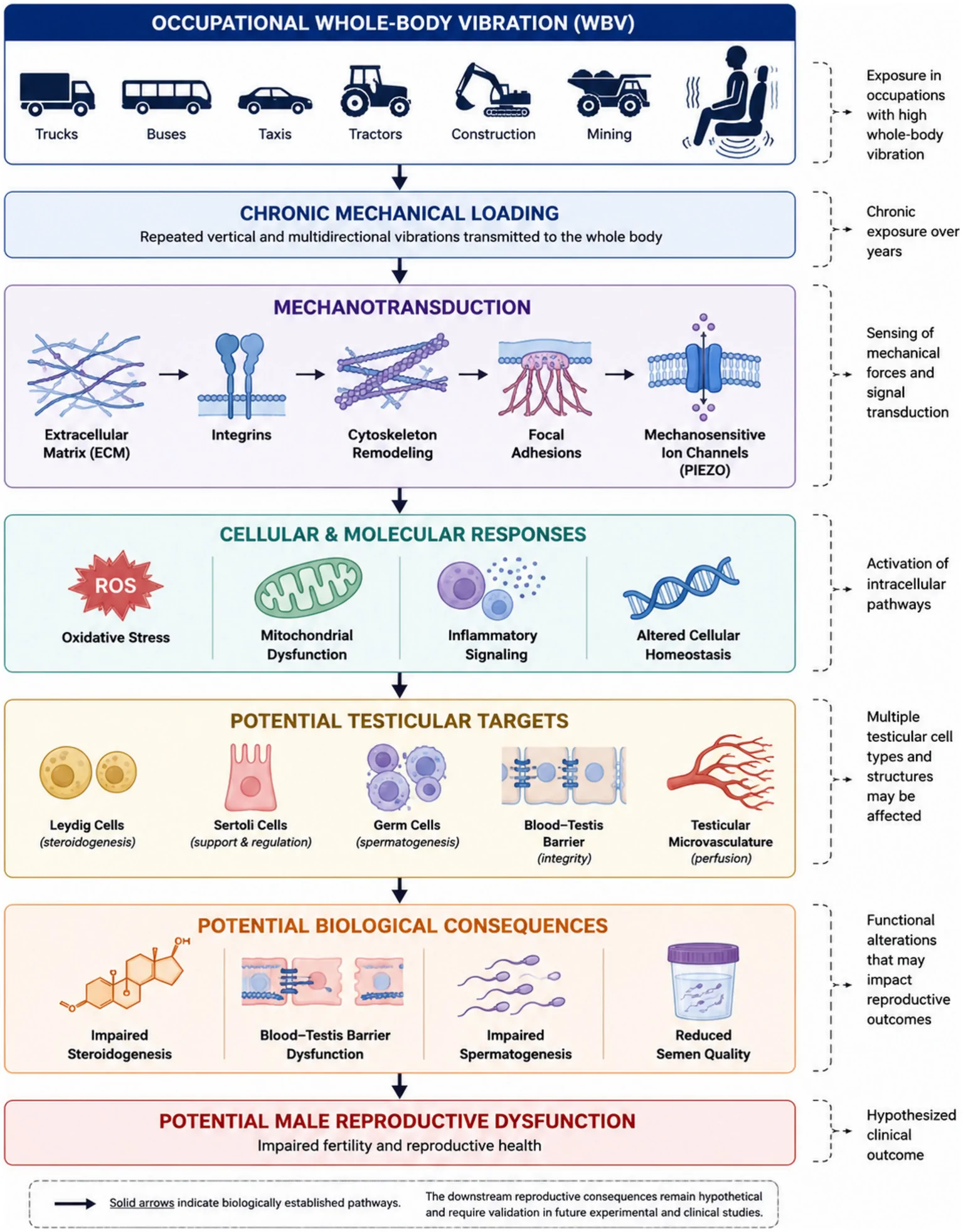
Proposed biological pathway linking occupational whole-body vibration to male reproductive dysfunction through mechanotransduction.

Recent studies have also highlighted the role of mechanosensitive ion channels, particularly the PIEZO family, in mediating cellular responses to mechanical stress (30). Furthermore, increased extracellular matrix stiffness has been shown to activate PIEZO1-dependent signaling, increase mitochondrial reactive oxygen species production, disrupt stem Leydig-cell homeostasis, and reduce testosterone synthesis in experimental models (31). While these findings were not derived from WBV exposure, they support the concept that chronic mechanical stimulation may directly influence testicular cellular function through mechanotransductive pathways. Specifically, PIEZO1 involvement in WBV-induced testicular responses remains hypothetical, as direct activation of this pathway by occupational WBV has not yet been demonstrated.

Collectively, current evidence suggests that mechanotransduction provides a biologically plausible framework linking chronic occupational mechanical exposure with downstream cellular responses in the testis. However, further studies are required to determine whether similar pathways are activated during long-term WBV exposure in humans.

### 4.2 Oxidative stress and mitochondrial dysfunction

Oxidative stress is widely recognized as a central mechanism contributing to male reproductive dysfunction. It occurs when the production of reactive oxygen species (ROS) exceeds the capacity of endogenous antioxidant systems, resulting in oxidative damage to cellular components (32–38).

Spermatozoa are particularly susceptible to oxidative injury because their plasma membranes contain high concentrations of polyunsaturated fatty acids and their cytoplasm contains relatively limited antioxidant defenses (32–35). Excessive ROS generation can promote lipid peroxidation, mitochondrial dysfunction, protein oxidation, DNA fragmentation, and impaired sperm motility, ultimately reducing fertilizing capacity (32–38).

Experimental studies of WBV have demonstrated increased oxidative stress markers together with reduced antioxidant enzyme activity in exposed tissues (32–35). Although most investigations have focused on non-reproductive organs, similar biological responses may occur within the testes because oxidative stress represents a common downstream consequence of chronic mechanical loading. In experimental reproductive models, oxidative stress has also been associated with germ-cell apoptosis, impaired Sertoli-cell function, and reduced testosterone production (32–38).

Mitochondria appear to play a central role in this process. Beyond their function in ATP production, mitochondria regulate cellular redox homeostasis, apoptosis, and intracellular signaling. Persistent mitochondrial dysfunction may therefore amplify oxidative injury and contribute to progressive impairment of spermatogenesis (32–38). Recent evidence linking mechanical stress to mitochondrial ROS production through PIEZO1 signaling further supports the possibility that mechanotransduction and oxidative stress are interconnected biological processes rather than independent mechanisms (31).

Although direct evidence from occupational WBV studies remains limited, oxidative stress represents one of the most biologically plausible pathways through which chronic mechanical exposure could adversely influence male reproductive health.

### 4.3 Testicular microcirculation and vascular dysfunction

Normal spermatogenesis depends on adequate testicular blood flow and microvascular integrity. The testicular microcirculation provides oxygen, nutrients, endocrine substrates, and metabolic exchange required for the continuous production and maturation of germ cells (16–18).

Chronic vibration exposure has been associated with vascular dysfunction in several organ systems, including endothelial injury, altered vasomotor regulation, impaired tissue perfusion, and microvascular remodeling (3–5). These vascular alterations are thought to result from repeated mechanical stress combined with oxidative and inflammatory responses.

Whether similar vascular changes occur within the testes remains uncertain. Nevertheless, the highly specialized testicular circulation may be particularly sensitive to chronic disturbances in endothelial function or local blood flow. Even modest reductions in tissue perfusion could increase oxidative stress, alter cellular metabolism, and compromise the microenvironment necessary for normal spermatogenesis (16–18).

Although direct evidence linking WBV to testicular vascular dysfunction is currently scarce, the available experimental data support further investigation of the testicular microvasculature as a potential target of chronic occupational mechanical exposure.

### 4.4 Blood-testis barrier integrity

The blood-testis barrier (BTB) is one of the most specialized epithelial barriers in the human body. Formed primarily by tight junctions between adjacent Sertoli cells, it creates a protected microenvironment that supports germ-cell development while limiting exposure to circulating toxins and immune cells (13–15).

Disruption of BTB integrity has been implicated in several causes of male infertility, including inflammation, oxidative stress, toxic exposures, and infection (13–15). Experimental studies indicate that oxidative injury and inflammatory mediators can alter junctional proteins, increase barrier permeability, and impair Sertoli-cell function, thereby compromising spermatogenesis (13–15).

Whether chronic WBV directly disrupts the BTB has not yet been established. However, because vibration exposure may promote oxidative stress, vascular dysfunction, and mechanotransductive signaling, secondary effects on BTB integrity are biologically plausible. Future experimental studies evaluating junctional proteins and barrier permeability following WBV exposure would help clarify this potential mechanism.

### 4.5 Endocrine dysfunction

Leydig cells are responsible for testosterone synthesis and play a critical role in maintaining spermatogenesis, secondary sexual characteristics, and male reproductive function. Testosterone production is regulated by the hypothalamic–pituitary-gonadal axis together with local paracrine signaling within the testis (17, 18).

Mechanical stress has recently been shown to influence Leydig-cell biology. Increased extracellular matrix stiffness has been associated with activation of PIEZO1 signaling, mitochondrial oxidative stress, depletion of stem Leydig cells, and reduced testosterone production in experimental models (31). Although these findings cannot be directly extrapolated to occupational WBV, they suggest that prolonged mechanical loading may influence endocrine function through mechanosensitive pathways.

In addition, oxidative stress has been shown to impair steroidogenesis by disrupting mitochondrial function and key enzymes involved in testosterone synthesis (32–38). Consequently, endocrine alterations may represent one component of a broader biological response to chronic mechanical exposure rather than an isolated mechanism.

### 4.6 Impaired spermatogenesis

Spermatogenesis is a highly coordinated process that depends on the integrity of germ cells, Sertoli cells, Leydig cells, adequate vascular supply, and an intact BTB. Disturbance of any of these components may compromise sperm production and quality (13–15, 32–38).

Experimental studies have reported alterations in sperm production, hormonal balance, oxidative stress markers, and testicular histology following prolonged WBV exposure (22). Human evidence is more limited but includes reports of reduced semen quality among workers with long-term occupational vibration exposure (19). Although these findings should be interpreted cautiously because of potential confounding factors and study heterogeneity, they support the need for further investigation.

Rather than acting through a single pathway, WBV may influence spermatogenesis through the combined effects of mechanotransduction, oxidative stress, vascular dysfunction, endocrine alterations, and possible disruption of the blood-testis barrier. At present, however, the relative contribution of each mechanism remains uncertain and requires confirmation in well-designed experimental and prospective human studies.

## 5 Current evidence linking whole-body vibration to male reproductive health

Although several biological mechanisms support a potential relationship between whole-body vibration (WBV) and male reproductive dysfunction, direct evidence remains relatively limited. Most available data originate from occupational observational studies and experimental animal models, with relatively few investigations specifically designed to evaluate reproductive outcomes. Consequently, the current evidence should be interpreted cautiously, while recognizing its biological plausibility and consistency across multiple mechanistic pathways.

### 5.1 Evidence from human occupational studies

Human studies evaluating the reproductive effects of occupational WBV remain scarce. Most investigations have focused on professional drivers and other workers exposed to prolonged vehicle-related vibration, occupations characterized by extended sitting, repetitive mechanical loading, and cumulative daily exposure (6–10).

One of the few studies specifically addressing male reproductive health reported that professional taxi drivers chronically exposed to WBV exhibited reduced semen quality compared with non-exposed controls (19). Observed alterations included deterioration in conventional semen parameters, suggesting that prolonged occupational vibration may adversely influence male reproductive function. Additional occupational evidence has examined WBV exposure in relation to male sex hormone levels (20), while a recent study examining multiple occupational exposures identified WBV among the variables associated with male fertility indicators (21). However, interpretation of these findings requires caution because multiple occupational and lifestyle factors—including prolonged sitting, heat exposure (42, 43), dietary patterns (44, 45), smoking, psychological stress, air pollution, and metabolic disorders—may also affect reproductive health and act as potential confounders.

Beyond reproductive outcomes, numerous epidemiological studies have consistently demonstrated systemic biological effects of chronic WBV, including musculoskeletal disorders, vascular dysfunction, neurological symptoms, and reduced physical performance (1, 3–10). These studies did not assess reproductive outcomes and therefore do not provide direct evidence of an effect of WBV on male fertility. However, they demonstrate biological responses to chronic WBV in non-reproductive systems and provide indirect context for investigating whether analogous responses may occur within reproductive tissues.

At present, the human evidence is insufficient to establish a causal relationship between WBV and male infertility. Nevertheless, the available occupational observations provide preliminary support for further investigation using prospective cohort studies with standardized reproductive outcome measures, including semen analysis according to current World Health Organization recommendations, hormonal assessment, and detailed exposure characterization (11, 39).

### 5.2 Evidence from experimental animal studies

Experimental animal models provide additional insight into the biological effects of WBV because exposure conditions can be more precisely controlled than in occupational settings. Several studies have demonstrated that prolonged vibration exposure may influence testicular structure and function, although experimental protocols differ considerably with respect to vibration frequency, acceleration, exposure duration, and animal species (22–24).

Reported findings include increased oxidative stress, reduced antioxidant activity, histological alterations within the seminiferous tubules, impaired spermatogenesis, hormonal changes, and increased germ-cell apoptosis following chronic WBV exposure (22). These observations are consistent with mechanisms involving oxidative injury, mitochondrial dysfunction, and altered cellular homeostasis discussed in previous sections.

Animal studies have also shown that mechanical stress can induce systemic inflammatory responses and vascular alterations that may indirectly affect reproductive tissues (3–5, 32–35). Although these findings cannot be directly extrapolated to humans, they support the biological plausibility that chronic mechanical exposure may influence testicular physiology through multiple interconnected pathways.

An important limitation of the experimental literature is the lack of standardized vibration protocols. Considerable variation exists in exposure frequency, magnitude, duration, and equipment, making direct comparison between studies difficult. Furthermore, relatively few investigations have evaluated mechanotransduction pathways, blood-testis barrier integrity, or mechanosensitive signaling in the context of WBV. These limitations highlight the need for more mechanistically focused experimental research.

### 5.3 Evidence integration

Taken together, the available evidence suggests a biologically plausible association between chronic occupational WBV exposure and male reproductive dysfunction, although current data remain insufficient to establish causality. Human studies are few in number and are susceptible to confounding, while experimental animal studies differ substantially in design and exposure conditions. Nevertheless, several independent lines of evidence converge on common biological pathways, including mechanotransduction, oxidative stress, mitochondrial dysfunction, vascular alterations, endocrine regulation, and impaired spermatogenesis (3–10, 13–15, 19, 22, 25–38).

Future investigations should prioritize well-designed prospective occupational cohorts with standardized vibration exposure assessment, comprehensive reproductive evaluation, and longitudinal follow-up. Integration of conventional semen analysis with hormonal profiling, oxidative stress biomarkers, advanced sperm function testing, and mechanotransduction-related molecular markers may help clarify whether chronic WBV contributes to clinically meaningful reproductive impairment. Such studies will be essential for determining whether reproductive health should be incorporated into future occupational WBV risk assessment and preventive strategies.

The available evidence discussed above is summarized in Table 1.

**Table 1:** Summary of direct, supportive, and mechanistic evidence relevant to occupational whole-body vibration and male reproductive health.

Reference	Study type/Evidence domain	Population/Model	WBV exposure	Main findings	Proposed mechanism(s)	Key limitations
Zarei et al. (19)	Human observational study— direct reproductive evidence	Professional taxi drivers	Chronic occupational WBV	Reduced semen quality compared with non-exposed controls	Not investigated	Cross-sectional design; multiple occupational confounders
Krajnak et al. (22)	Experimental animal study— direct reproductive evidence	Rat model	Controlled WBV exposure	Histological alterations, impaired spermatogenesis, hormonal changes, increased oxidative stress	Oxidative stress, cellular injury	Animal model; limited clinical translation
Penkov and Tzvetkov (23)	Narrative review— supportive reproductive evidence	Occupational WBV	Chronic vibration exposure	Suggested potential adverse reproductive effects	Biological plausibility	Limited primary evidence
Mazlomi et al. (24)	Systematic review—supportive reproductive evidence	Occupational populations	WBV exposure	Identified vibration as a potential occupational reproductive risk factor	Multiple proposed biological pathways	Heterogeneity among included studies
Ingber (25), Vogel and Sheetz (26), Jaalouk (27), Ingber (28), Ingber (29)	Experimental and mechanobiology studies— indirect mechanistic evidence	Cellular and experimental models	Mechanical loading	Mechanical stimuli regulate intracellular signaling through mechanotransduction	ECM, integrins, cytoskeleton, focal adhesions	Not specific to WBV or testicular tissue
Xiao (30)	Mechanobiology review— indirect mechanistic evidence	Cellular models	Mechanical stimulation	PIEZO channels mediate cellular responses to mechanical forces	PIEZO-dependent mechanotransduction	Limited reproductive-specific evidence
Zhang et al. (31)	Experimental study— indirect mechanistic evidence	Stem Leydig-cell model	Increased extracellular matrix stiffness	PIEZO1 activation increased mitochondrial ROS and reduced testosterone synthesis	Mechanotransduction, mitochondrial dysfunction	Not a WBV exposure model
Aitken et al. (35), Agarwal et al. (32), Aitken and Roman(33), Tremellen (34), Skakkebaek et al. (36), Sharpe (37), Sengupta et al. (38)	Reviews and experimental studies— indirect mechanistic evidence	Male reproductive system	Various experimental models	Oxidative stress impairs sperm function, DNA integrity, and steroidogenesis	ROS generation, mitochondrial dysfunction	Not specific to WBV
Cheng and Mruk (13), Stanton (14), França et al. (15)	Reviews and experimental studies— indirect mechanistic evidence	Testicular biology	Various experimental models	Blood-testis barrier integrity is essential for normal spermatogenesis	Sertoli-cell dysfunction, barrier disruption	No direct WBV evidence
Ergün et al. (16), Ergün et al. (17), Heinrich and DeFalco (18)	Reviews and experimental studies— indirect physiological evidence	Testicular physiology	Various experimental models	Testicular microcirculation is essential for maintaining testicular homeostasis	Vascular regulation	Limited WBV-specific evidence
Mohammadi et al. (20)	Human occupational study— direct endocrine evidence	162 male workers in a factory manufacturing automobile parts	Occupational WBV and noise exposure	WBV showed the greatest effect on testosterone in logistic regression; however, only the noise–testosterone association was statistically significant in correlation analysis	Endocrine alterations	Concurrent noise exposure; observational design; WBV-specific associations were not consistently significant across analyses
Mohammadi et al. (21)	Human occupational study— direct reproductive evidence	80 male workers in a factory manufacturing automobile parts	Multiple occupational exposures, including WBV, noise, heat stress, and electric and magnetic fields	WBV was evaluated among predictors of male reproductive indicators, including sex hormones and sperm parameters, using machine-learning models	Multifactorial occupational pathways	Multiple simultaneous exposures; observational associations do not establish causality

## 6 Discussion

Despite decades of research on occupational whole-body vibration (WBV), its potential impact on male reproductive health has received remarkably little attention. Existing literature has primarily focused on musculoskeletal disorders, spinal degeneration, neurological symptoms, and vascular complications (1, 2, 6–10). This review does not claim that WBV is an established cause of male infertility. Rather, it highlights that current evidence from occupational medicine, reproductive biology, and mechanobiology collectively suggests a biologically plausible association that deserves greater scientific attention.

The principal contribution of this review is the integration of evidence that has previously been considered largely in isolation. Human occupational studies have reported reduced semen quality among workers chronically exposed to WBV (19) and have examined sex-hormone and fertility indicators in workers exposed to WBV alongside other occupational factors (20, 21). Experimental studies have demonstrated reproductive alterations following prolonged vibration exposure (22–24), and recent advances in mechanobiology have shown that mechanical stimuli can regulate cellular behavior through mechanotransduction pathways involving the extracellular matrix, cytoskeleton, integrins, and mechanosensitive ion channels (25–31). To our knowledge, these observations have not previously been synthesized into a unified framework to explain how chronic occupational mechanical loading may influence male reproductive function.

A second important contribution is the application of mechanotransduction to occupational reproductive medicine. Mechanotransduction is well established in bone biology, vascular physiology, and tissue remodeling, yet its potential relevance to the testis has received comparatively little attention (25–29, 46). Recent mechanobiological studies of the male reproductive tract further suggest that mechanical forces and luminal fluid dynamics may regulate reproductive tissue homeostasis beyond the testis, supporting the broader relevance of mechanobiological signaling within the male reproductive system (46). Recent evidence demonstrating that mechanical stiffness influences Leydig-cell homeostasis through PIEZO1-dependent signaling provides proof that testicular cells respond to mechanical stimuli (31). Although these findings were not obtained from WBV models, they provide a biologically plausible mechanism through which chronic vibration exposure could influence testicular physiology. This perspective shifts the discussion beyond descriptive occupational observations toward a mechanistic hypothesis that can be experimentally tested.

Another novel aspect of this review is the integration of multiple downstream biological pathways into a single conceptual model. Rather than considering oxidative stress, vascular dysfunction, endocrine alterations, blood-testis barrier disruption, and impaired spermatogenesis as independent observations, we propose that these mechanisms may represent interconnected biological responses initiated or amplified by chronic mechanical loading. Such an integrated perspective may help explain why reproductive dysfunction, when present, is unlikely to result from a single pathogenic pathway.

Based on this synthesis, we introduce the concept of Testicular Ergonomics. We do not propose this as a new disease entity or a replacement for existing occupational health principles. Instead, Testicular Ergonomics is presented as a conceptual framework that extends traditional ergonomics by considering the potential influence of chronic occupational mechanical exposure on male reproductive health. Similar to how ergonomics seeks to minimize musculoskeletal injury through optimized workplace design, this concept encourages future research into whether reproductive organs should also be considered when evaluating long-term occupational vibration exposure. In practical terms, this framework may include established WBV-reduction measures such as vibration-attenuating seating and suspension systems, including frequency-dependent attenuation where technically appropriate, equipment maintenance, optimization of work–rest schedules and micro-breaks, and standardized exposure assessment according to ISO 2631-1. These measures are proposed to reduce occupational WBV exposure generally (11, 40, 41). However, their specific effectiveness in preventing male reproductive effects remains to be established. At present, this framework should be regarded as hypothesis-generating rather than evidence-confirming. The proposed concept of Testicular Ergonomics is illustrated in Figure 2.

**Figure 2:**
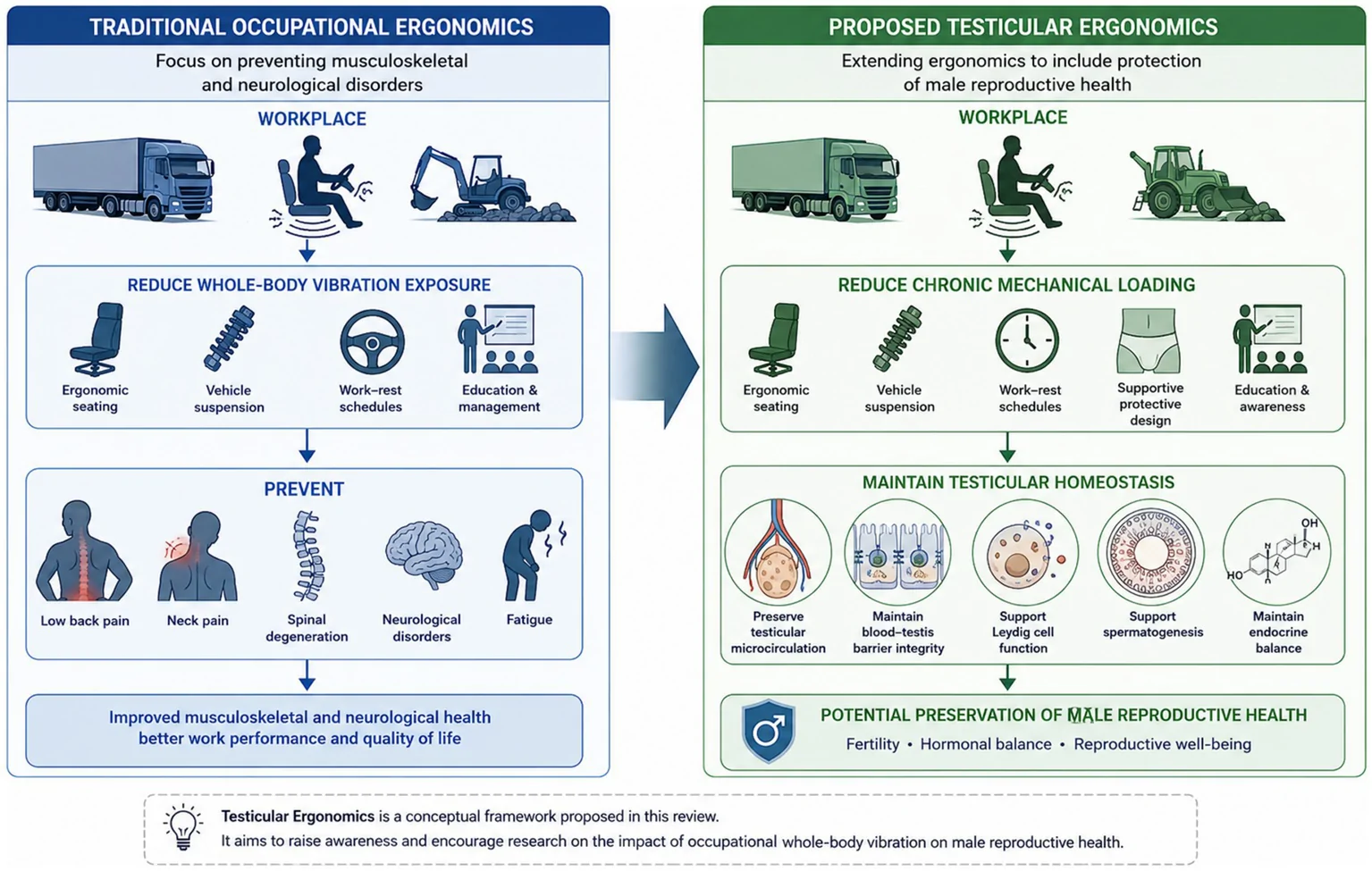
Proposed conceptual framework of Testicular Ergonomics for preserving male reproductive health during occupational whole-body vibration exposure.

The review also identifies several important knowledge gaps. Most available human studies are cross-sectional and involve relatively small populations, while experimental investigations use heterogeneous vibration protocols, making direct comparison difficult (19, 22–24). Furthermore, no studies have systematically evaluated mechanotransduction signaling, PIEZO activation, blood-testis barrier integrity, or testicular microvascular function following occupational WBV exposure. These limitations highlight important opportunities for future translational research.

Future studies should combine standardized WBV exposure assessment according to ISO 2631 with comprehensive reproductive evaluation, including semen analysis following current World Health Organization recommendations, hormonal profiling, oxidative stress biomarkers, and molecular markers of mechanotransduction (11, 39, 47). Advances in testicular bioengineering also emphasize the importance of understanding the mechanical and cellular microenvironment of the testis, providing additional support for investigating mechanobiological pathways in occupational reproductive research (47). Such multidisciplinary approaches may determine whether chronic WBV represents an independent occupational risk factor for male reproductive dysfunction or whether the observed associations are primarily explained by coexisting occupational and lifestyle factors.

Overall, the present review expands the discussion of occupational WBV beyond its traditional musculoskeletal focus by integrating occupational medicine, reproductive biology, and mechanobiology into a single conceptual framework. Although definitive clinical evidence remains limited, this synthesis provides a rationale for future mechanistic and prospective human studies and introduces Testicular Ergonomics as a framework to guide research at the intersection of occupational vibration and male reproductive health.

If validated by future experimental and epidemiological studies, Testicular Ergonomics could contribute to the development of occupational guidelines that integrate reproductive health into vibration exposure assessment.

## 7 Conclusion

Occupational whole-body vibration has traditionally been investigated in relation to musculoskeletal and neurological disorders, whereas its potential effects on male reproductive health have received comparatively little attention. Although current evidence remains limited and does not establish causality, available occupational, experimental, and mechanobiological findings collectively support further investigation of this relationship.

This review brings together evidence from occupational medicine, reproductive biology, and mechanotransduction to provide a multidisciplinary perspective on how chronic mechanical loading may influence testicular function. By introducing the concept of Testicular Ergonomics, we propose a hypothesis-generating framework that encourages consideration of male reproductive health within future occupational vibration research rather than suggesting changes to current clinical or occupational practice.

Future studies integrating standardized vibration exposure assessment with reproductive and molecular outcomes will be essential to determine whether chronic occupational whole-body vibration represents a clinically relevant reproductive risk and to inform evidence-based preventive strategies. Prospective occupational studies should also evaluate whether established WBV-reduction measures, including vibration-attenuating seating and suspension systems, equipment maintenance, and optimized work–rest schedules, can reduce potential reproductive risks.
